# Brain sparing and the blood brain barrier—bridging the preclinical to clinical gap

**DOI:** 10.1038/s41390-025-04479-y

**Published:** 2025-10-02

**Authors:** Tegan A. White, Suzanne L. Miller

**Affiliations:** 1 The Ritchie Centre, Department of Obstetrics and Gynaecology, Monash University, Clayton, VIC Australia; 2 Hudson Institute of Medical Research, Clayton, VIC Australia

**Commentary Article** on *Gestational hypoxia increases blood-brain barrier permeability in the neonatal cerebral cortex of guinea pigs*, by E. Figueroa et al.

The brain, and particularly the fetal brain, is the most highly metabolically active and energy-expensive organ in the body, with the developing fetal brain reliant on oxygen and nutrients supplied across the placenta.^1^ Pathological reductions in fetal oxygen supply during pregnancy are relatively common, caused by acute stress such as during labor and contractions, or induced chronically secondary to placental insufficiency. In the case of placental insufficiency and chronic fetal hypoxemia, the fetus decreases fetal growth, resulting in fetal growth restriction (FGR). The fetus also mounts a physiological response to hypoxemia, redistributing blood flow towards the brain at the expense of non-essential tissue beds—this adaptation is called brain sparing.^2^ This brain sparing response is considered a survival mechanism, ensuring adequate oxygen supply to the brain during fetal compromise and, indeed when prolonged, this adaptation protects brain growth relative to other organs resulting in asymmetric FGR (the head is relatively larger than other organs).^2,3^ Clinically, fetal brain sparing is detected via Doppler ultrasound as vasodilatation of the middle cerebral artery (MCA) and/or a reduction in the cerebroplacental ratio (CPR).^4,5^ Accumulating evidence indicates that brain sparing may be associated with elevated risk of adverse neurodevelopmental outcome,^4,5^ yet the pathophysiology linking prolonged cerebral vasodilation, brain sparing and altered brain development at the cellular level remains largely uncharacterized. Here we provide a commentary on the work of Figueroa et al.^6^ who examined how chronic fetal hypoxia and brain sparing affect cerebrovascular development.

Figueroa et al.^6^ utilize a preclinical guinea pig model of chronic fetal hypoxia and brain sparing to examine the integrity of the blood-brain barrier (BBB) within the cerebral cortex and white matter of the offspring after birth. A notable strength of this work is the application of fetal Doppler ultrasound to confirm MCA vasodilatation in late gestation hypoxic guinea pigs, complemented with functional analysis of carotid endothelial reactivity after birth, and molecular and cellular interrogation of key cerebrovascular components. This is a considered experimental design that allows insight into the adaptations of cerebral vascular cells that accompany clinical detection of MCA vasodilatation. As we would expect, late gestation hypoxic guinea pig fetuses demonstrated reduced cerebrovascular resistance and redistribution of cardiac output towards the brain, indicated by decreased MCA pulsatility index and decreased CPR, as is observed clinically in response to placental dysfunction and FGR.

Brain sparing was confirmed at birth in guinea pig offspring, with Figueroa et al. showing that chronic antenatal hypoxia induced an asymmetric growth phenotype typical of FGR, and subsequent analyses were then undertaken to assess cerebrovascular structure and function.^6^ Firstly, the functional consequences of chronic antenatal hypoxia were assessed using carotid artery myography on vessels collected after birth. Results showed enhanced endothelial and smooth muscle-mediated vasodilation in carotid arteries exposed to chronic hypoxia during gestation. This is important, indicating that an adverse antenatal environment predisposes to altered vascular reactivity after birth. FGR infants are often born preterm and exposed to multiple stressors in the newborn period, with significantly increased rates of morbidity.^7^ A recent study that followed up FGR lambs for 4 weeks after birth showed that early life differences in femoral artery vasoreactivity at newborn age were not sustained, and indeed were reversed by 4 weeks of age.^8^ When taken together, the results of these preclinical guinea pig and sheep studies are very useful to expand our understanding regarding the clinical consequences of brain sparing, and serve to highlight the complex relationship between cardiovascular adaptations and neurological risk, and emphasize that these systems do not respond in isolation.

For structural analysis within the brain, Figueroa et al. paid close attention to the BBB and component cells of the neurovascular unit (NVU), postulating that prolonged hypoxia and vasodilatation are likely to disrupt the interactions between cells that form the physical barriers between the brain’s circulation and brain parenchyma.^6^ A strength of this study is a consistent demonstration of BBB disruption in the neonatal brain exposed to hypoxia, across multiple lines of analysis, from gene transcripts to proteins, and supported by immunohistochemical analysis of brain tissue, providing compelling and robust evidence. The tight junction proteins of the BBB are essential for maintaining the integrity of the BBB, with tight junctions occupying the spaces between vascular endothelial cells, regulating transcellular movement of substances between the circulation and the brain tissue. Claudin-1, -3, -5, and -12, together with occludin, are integral tight junction proteins that directly mediate permeability across the BBB.^9^ Of the tight junctions, it is claudin-5 in particular that is recognized as essential for maintaining BBB integrity,^9^ with downregulation or loss of claudin-5 strongly associated with the pathogenesis of brain injury in multiple disorders.^10^ In the present study, Figueroa et al. observed decreases in both claudin-5 and claudin-12 gene and protein expression within cortical gray matter tissue, followed up with corroboration using immunohistochemical staining.^6^ This confirms that claudin-5 is also susceptible to change with chronic hypoxia in the developing brain, with previous reports of reduced claudin-5 following acute severe perinatal hypoxia.^11^ The earlier study by Jithoo and colleagues showed that a reduction in claudin-5 protein became more pronounced over time, between 6 h and 72 h after hypoxic insult, and claudin-5 expression was highly correlated with the presence of brain microbleeds.^11^ Thus, this loss of critical tight junction proteins is accompanied by a cascade of barrier dysfunctions, evidenced by increased permeability and albumin extravasation into gray matter, underscoring the consequences of these molecular disruptions.

The work of Figueroa et al.^6^ provides novel evidence linking brain sparing, reduced endothelial tight junctions and a “leaky” BBB in the FGR brain, however the clinical implications remain unresolved. The results presented here would predict that neonates with brain sparing and FGR predispose the infant to a greater likelihood of brain microbleeds or intraventricular hemorrhage (IVH), but a recent large clinical study provides clear evidence that this is not the case in growth-restricted infants born very preterm.^12^ Piscopo et al. showed that in very preterm infants, the presence of SGA or FGR is associated with a *reduced* rate of IVH, speculating that growth restricted infants may have compensatory mechanisms of brain sparing adaptation with cerebrovascular remodeling, or improved outcome may reflect how FGR infants are managed at birth.^12^ Future work should investigate whether a clinical correlation exists between severity of growth restriction, the degree of brain sparing and subsequent fragility of the BBB, as observed in the present study. Understanding this relationship is vital, as tight junction dysfunction may serve as a biomarker for brain injury in FGR, and BBB disruption is both a consequence of injury and plays a key factor in perpetuation of injury.^11^ Notably, BBB-related proteins can be detected in the maternal circulation,^13^ potentially providing an early in utero indication of which infants are at increased risk for cerebrovascular compromise and subsequent adverse neurological outcomes postnatally.

The expression “brain sparing” infers that this adaptive physiological response spares the brain from injury. This is true in the sense that fetal hypoxia induces a haemodynamic redistribution of cardiac output towards the brain to prefer cerebral oxygen delivery. But conversely, accumulating evidence indicates that the antenatal presence of brain sparing exacerbates neurodevelopmental deficits in human infants born growth restricted.^4,5^ Preclinical studies increase our understanding of the pathophysiology linking chronic fetal hypoxia, brain sparing and altered brain development at the cellular level. The work highlighted in this commentary adds new insight that the brain sparing response is mediated by chronic cerebral vasodilatation that necessitates cerebrovascular remodeling, and in turn this adversely modifies vascular reactivity after birth. Chronic antenatal hypoxia and cerebrovascular remodeling reduce critical endothelial tight junction proteins within cerebral gray matter, decreasing the integrity of the blood brain barrier and increasing the likelihood of blood-borne infiltrates entering and injuring brain tissue. Finally, results presented by Figueroa et al.^6^ stress the importance of the tight junction protein claudin-5 in the FGR brain, with claudin-5 showing future promise as both a biomarker of cerebrovascular vulnerability in FGR and therapeutic neuroprotective target. These findings bridge the gap between clinical and preclinical consequences of brain sparing, showing that the clinical ultrasound diagnosis of brain vasodilatation is associated with specific sub-cellular deficits in tight junction proteins, which are likely to underpin an increased susceptibility to cerebrovascular dysfunction and brain injury after birth.
